# Nutrient Stoichiometry Shapes Microbial Community Structure in an Evaporitic Shallow Pond

**DOI:** 10.3389/fmicb.2017.00949

**Published:** 2017-05-30

**Authors:** Zarraz M.-P. Lee, Amisha T. Poret-Peterson, Janet L. Siefert, Drishti Kaul, Ahmed Moustafa, Andrew E. Allen, Chris L. Dupont, Luis E. Eguiarte, Valeria Souza, James J. Elser

**Affiliations:** ^1^School of Life Sciences, Arizona State University, TempeAZ, United States; ^2^School of Earth and Space Exploration, Arizona State University, TempeAZ, United States; ^3^Department of Statistics, Rice University, HoustonTX, United States; ^4^J. Craig Venter Institute, La JollaCA, United States; ^5^Department of Biology and Biotechnology Graduate Program, American University in CairoNew Cairo, Egypt; ^6^Integrative Oceanography Division, Scripps Institution of Oceanography, University of California, San Diego, La JollaCA, United States; ^7^Departamento de Ecología Evolutiva, Instituto de Ecología, Universidad Nacional Autónoma de MéxicoCiudad de México, Mexico; ^8^Flathead Lake Biological Station, University of Montana, PolsonMT, United States

**Keywords:** stoichiometry, community structure, beta diversity, bacteria, algae, rRNA gene copy number, growth rate hypothesis

## Abstract

Nutrient availability and ratios can play an important role in shaping microbial communities of freshwater ecosystems. The Cuatro Ciénegas Basin (CCB) in Mexico is a desert oasis where, perhaps paradoxically, high microbial diversity coincides with extreme oligotrophy. To better understand the effects of nutrients on microbial communities in CCB, a mesocosm experiment was implemented in a stoichiometrically imbalanced pond, Lagunita, which has an average TN:TP ratio of 122 (atomic). The experiment had four treatments, each with five spatial replicates – unamended controls and three fertilization treatments with different nitrogen:phosphorus (N:P) regimes (P only, N:P = 16 and N:P = 75 by atoms). In the water column, quantitative PCR of the 16S rRNA gene indicated that P enrichment alone favored proliferation of bacterial taxa with high rRNA gene copy number, consistent with a previously hypothesized but untested connection between rRNA gene copy number and P requirement. Bacterial and microbial eukaryotic community structure was investigated by pyrosequencing of 16S and 18S rRNA genes from the planktonic and surficial sediment samples. Nutrient enrichment shifted the composition of the planktonic community in a treatment-specific manner and promoted the growth of previously rare bacterial taxa at the expense of the more abundant, potentially endemic, taxa. The eukaryotic community was highly enriched with phototrophic populations in the fertilized treatment. The sediment microbial community exhibited high beta diversity among replicates within treatments, which obscured any changes due to fertilization. Overall, these results showed that nutrient stoichiometry can be an important factor in shaping microbial community structure.

## Introduction

The absolute and relative supplies of nitrogen (N) and phosphorus (P) in the environment have a major influence on the diversity of species at macro- and microscopic scales (Elser et al., 2005b; Leflaive et al., 2008). Hence, the availabilities and ratios of key limiting nutrients, such as N and P, have been suggested to be fundamental in understanding microbial diversity (Torsvik et al., 2002; Newton et al., 2011; Groszkopf and Soyer, 2016). Indeed, studies involving natural gradients or *in situ* experimental manipulation across different time scales and environments show that nutrient availability affects biodiversity. When responses are observed, effects range from little impact to large alterations in community structure, reflected as changes in species richness often accompanied by shifts in dominance/evenness (Claire Horner-Devine et al., 2003; Hewson et al., 2003; Bowen et al., 2011; Van Horn et al., 2011; Logue et al., 2012; Soininen and Meier, 2014). Microbial community responses to nutrients are likely rooted in the metabolic diversity and ecological strategies of the responsive taxa (Carbonero et al., 2014); therefore, attention should also be given to the ability of individual taxonomic groups or specific taxa to access and use nutrient inputs (Haukka et al., 2006; Nelson and Carlson, 2011; Peura et al., 2012; Corman et al., 2016). However, our ability to predict how various microbial taxa respond to nutrient enrichment is still limited.

More recently, the theory of biological stoichiometry had been used to integrate evolutionary biology and ecosystem ecology in both macro- and microbiology (Elser, 2003; Hall et al., 2011). Biological stoichiometry provides a mechanistic theory that links cellular and biochemical features of biota with the environmental constraints imposed by the supplies of multiple limiting nutrients, especially N and P (Elser et al., 2006; Hillebrand et al., 2014). Thus, understanding variation in biomass carbon:nitrogen:phosphorus (C:N:P) stoichiometry provides an avenue to understand community responses to nutrient supply. In particular, the Growth Rate Hypothesis, GRH (Elser et al., 2000), postulates that an organism’s C:N:P stoichiometric requirements are dependent on its growth rate because elevated growth rate depends on increased production and maintenance of P-rich ribosomes. Various field and laboratory studies have shown that growth, RNA content (percent of dry mass), and biomass P content are often tightly coupled within and across species, and especially under physiological P limitation (Elser et al., 2003; Makino and Cotner, 2004; Klausmeier et al., 2007; Chan et al., 2012; Hessen et al., 2013). Furthermore, it has been proposed that maximum growth rate, as a key life history parameter, and ribosome production capacity have a genomic basis in the multiplicity of ribosomal RNA operons (rRNA gene copy number) (Weider et al., 2005; Stoddard et al., 2014; Roller et al., 2016). This is consistent with studies that have shown that microbes with fewer copies of rRNA genes tend to be more competitive in oligotrophic or low nutrient conditions due to their high efficiency in resource use, whereas copiotrophs with higher rRNA gene copy numbers tend to respond more rapidly and are adapted to high nutrient supply and episodic availability (Klappenbach et al., 2000; Lauro et al., 2009; Nemergut et al., 2016; Roller et al., 2016). Thus, it is expected that P availability should have an especially significant effect on fast-growing taxa and/or those with elevated rRNA gene copy number and, consequently, overall community structure. However, the rRNA gene copy number hypothesis has not yet been experimentally tested under field conditions. Hence, assessing impacts of nutrient supply and stoichiometry in P-limited ecosystems may help in understanding the underlying mechanism of how nutrients shape microbial community structure.

Microbe-dominated, P-limited aquatic ecosystems suitable for such tests are found in the Cuatro Ciénegas basin (CCB), an oasis of oligotrophic springs in the Chihuahuan desert in the state of Coahuila in northwest Mexico. CCB contains diverse and often endemic microbes and macroorganisms (Souza et al., 2006, 2012) that persist in aquatic ecosystems with very low available P concentrations as well as strong stoichiometric imbalance with nitrogen (N:P ratios commonly exceed 100:1 by atoms). Hence, these ecosystems are strongly limited by P but with secondary impacts of N (Elser et al., 2005a; Lee et al., 2015), making them an excellent system to study the effects of nutrient ratios and to evaluate the GRH (Elser et al., 2000, 2005a,b). In this study, we use spatially replicated *in situ* mesocosms to investigate the effects of altered nutrient stoichiometry on bacterial and microbial eukaryotic communities in Lagunita, an evaporative pond in the CCB. We previously reported large effects of this fertilization on nutrient pools, biomass concentrations, and biomass C:N:P stoichiometry (Lee et al., 2015). Here, we describe the impact of nutrient stoichiometry on microbial community composition using high-throughput pyrosequencing of bacterial and eukaryotic small subunit (SSU) rRNA genes.

## Materials and Methods

### Study Location and Experimental Design

The *in situ* nutrient enrichment experiment was conducted during summer 2011 in a small (∼12 m × 4 m on average) shallow (<0.33 m) evaporitic pond, Lagunita (26.84810° N, 102.14160° W), lateral to the main Churince flow system in CCB in the state of Coahuila, Mexico. The Churince system is located at the western region of CCB and is dominated by gypsum-rich sediments. Lagunita is characterized by low P concentrations (PO_4_^3-^ as low as 0.1 μM) but relatively high concentrations of inorganic N and thus high N:P ratios (>200:1 by atoms) (Lee et al., 2015). Lagunita is also low in macrophyte abundance, reducing the potential confounding factor of plant–microbe interactions.

The mesocosm experiment was described in detail in Lee et al. (2015). Briefly, the mesocosms consisted of clear plastic cylinders (40-cm diameter) that were inserted into the pond sediments and extended above the water surface by 20 cm. Five replicated blocks of four treatments were established along an east–west transect of the pond. The treatments were unenriched (U), P-only (P), N and P at N:P = 16 (NP16), and N:P = 75 (NP75). P was applied as KH_2_PO_4_ while N was applied as NH_4_NO_3_. Nutrients were re-applied every 3–4 days to maintain a soluble reactive phosphorus (SRP) concentration of 1 μM (an approximate 16-fold increase over initial SRP concentration of 0.06 ± 0.02 μM) and appropriate N:P ratio. This fertilization regime was maintained for 21 days. Pre-fertilization values of total P and total N in the water column were 1.79 ± 0.20 μM and 187 ± 8.58 μM, respectively. After 3 weeks of periodic fertilization, P addition increased total P in fertilized treatments by >3.5-fold, while total N in the NP16 and NP75 mesocosms increased by >40% and >3-fold, respectively (Lee et al., 2015).

### Sample Collection and DNA Extraction

Water column and sediment samples were collected on day 21. Water column samples were collected by filtering 120 – 140 mL of water onto sterile GF/F filters (0.7-μm nominal pore size, Whatman, Piscataway, NJ, United States). GF/F filters were used in order to capture sufficient amount of biomass for DNA extraction. Sediment samples were collected by scooping the top ∼2 mm of the surface with a plastic spatula into cryovials. Both water column and sediment samples were flash-frozen in liquid N_2_ and stored at -80°C until extraction.

DNA was extracted from water column samples using the MO BIO PowerWater DNA Isolation kit, with one modification (Mo Bio Laboratories, Carlsbad, CA, United States). The volume of PW1 solution was increased to 1.5 mL due to the high absorbency of the GF/F filters. The DNA extraction method for sediment samples was modified from Purdy (2005). Briefly, frozen sediment was thawed by centrifugation to remove pore water. The sediment was then transferred into a MO BIO Bead tube for mechanical lysis in a FastPrep^®^-24 (MP Biomedicals, Solon, OH, United States) in a solution of Tris-buffered phenol and acid washed polyvinylpolypyrrolidone (PVPP). The extracted DNA was purified by column filtration through Sephadex G-50 (Sigma, St. Louis, MO, United States) and Bio-Gel HTP hydroxyapatite (Bio-Rad, Hercules, CA, United States) followed by ethanol precipitation. DNA yield and quality were assessed by Picogreen assay (Life Technologies, Carlsbad, CA, United States) and PCR amplification as described below.

### qPCR Analysis

A measure of 16S rRNA gene copy number of water column samples was determined using quantitative PCR (qPCR) with primers targeting the V6 region of the gene (967F and 1046R) (Huber et al., 2009). qPCR was performed on 10-fold dilutions of DNA extracts. A standard curve was constructed from plasmid containing a cloned V6 region of 16S rRNA gene from *Bacillus* sp. m3-13 and ranged from 9.5 × 10^2^ to 9.5 × 10^7^ copies μL^-1^ with efficiencies of 98% or higher. Triplicate 10-μL qPCR reactions were performed for each sample in a PikoReal 96 Real-Time PCR system (Thermo Scientific, Inc., Waltham, MA, United States). Each reaction contained 1 μl DNA template, 1X DyNAmo ColorFlash SYBR Green qPCR master mix and 500 nM of each primer. The cycling parameters were as follows: (1) initial denaturation at 95°C for 7 min, (2) 40 cycles of 95°C for 30 s, 57°C for 30 s, and 72°C for 30 s with fluorescence capture after extension, (3) final extension at 72°C for 30 s, and (4) melt curve analysis from 55 to 95°C. The qPCR results were used to calculate the abundance 16S rRNA gene copies as gene copies per ml water (copies mL^-1^). Gene copy number was then divided by the number of cells per ml water (cells ml^-1^) obtained from epifluorescence microscopy.

Differences in 16S rRNA gene copy number (normalized to bacterial cell counts) between the unenriched and fertilized treatments were assessed by analysis of variance (ANOVA) followed by Tukey’s HSD *post hoc* tests to compare individual groups. Only water samples were analyzed due to interferences with qPCR of sediment-derived DNA.

### SSU rRNA Gene Sequence Processing and Analyses

The 16S rRNA gene V4-V5 region and 18S rRNA gene V4 region was PCR amplified from all DNA samples with barcoded primers. The 16S forward primer combined the 357F primer (CCTACGGGAGGCAGCAG) with the Titanium B adapter (CCTATCCCCTGTGTGCCTTGGCAGTCTCAG). The 16S reverse primer combined the 926R primer (CCGTCAATTCMTTTRAGT) with the Titanium A adapter (CCATCTCATCCCTGCGTGTCTCCGACTCAG) with a 10 nucleotide barcode in between. The primer set 357F/926R targets >96% of bacteria; however, it does not capture 16S rRNA genes from Epsilonproteobacteria and Archaea when tested using SILVA TestPrime (Klindworth et al., 2013; see Supplementary Figure 3 in Dupont et al., 2014). The 18S primers were similarly designed but use the EukV4F (CCAGCASCYGCGGTAATTCC) and EukV4R (ACTTTCGTTCTTGATYRA) primers. The amplicons were checked by gel electrophoresis and submitted to JCVI for sequencing. Amplicons for each sample were quantified by qPCR and pooled prior to pyrosequencing using the 454 Titanium pipeline (454 Life Sciences, Branford, CT, United States). The sequences are available at NCBI under the accession number PRJNA311559.

Reads were de-multiplexed according to the barcodes and trimmed of barcodes and adapters. Taxonomic affiliation of the aligned reads was determined using the SILVA database by BLAST search to generate phylotypes, which is specific to the genus level (Quast et al., 2013). All sequences that passed the sequence processing screen, including those that could only be classified as Bacteria or Eukarya, were included in downstream analyses. To compare the difference in community structure, relative abundance of each phylotype was calculated as percent sequence abundance for each library. The 50 most abundant phylotypes among all the samples are presented as a heatmap using the ggplot2 package v 2.1.0 in R (Wickham, 2009).

**FIGURE 1:**
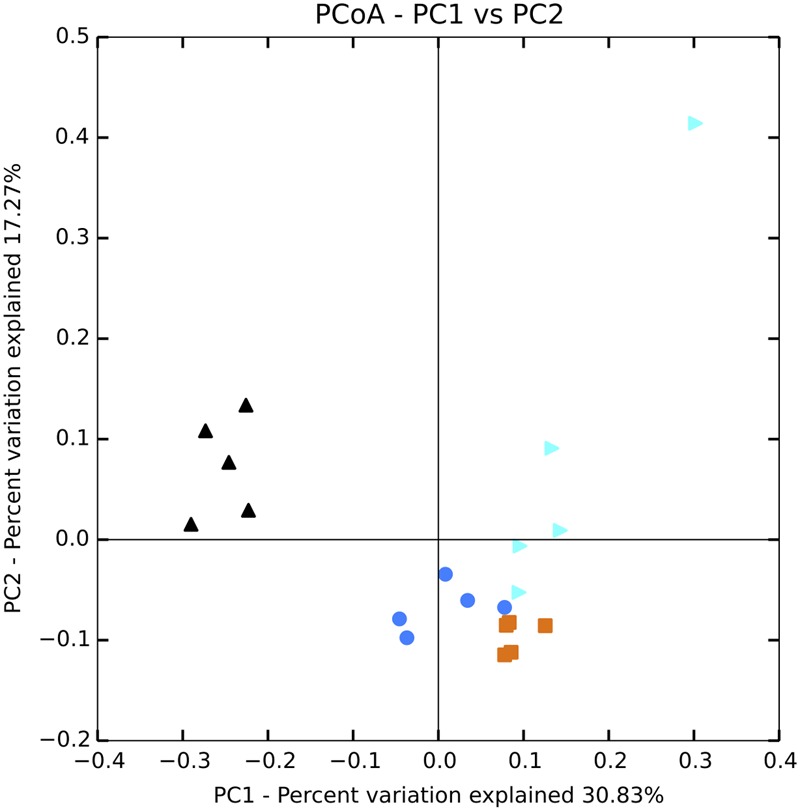
**Principal coordinates analysis (PCoA) plot of Bray–Curtis distances for bacterial communities in the water column.** Black = unenriched, orange = P-only, light blue = NP16, dark blue = NP75.

To determine if fertilization had significant effects on planktonic and sediment community composition or structure, several statistical approaches were applied on normalized libraries using the cumulative sum scaling method (Paulson et al., 2013). Phylotype data were used for all statistical analyses to allow the same method of sequence analysis for both bacterial and eukaryotic sequence libraries. The more conservative phylotype-based approach was selected because the sequence similarity value for operational taxonomic unit (OTU) calling of 18S rRNA gene sequences into OTUs is still debatable due to the heterogenous evolution rate for this gene among different eukaryotic taxa (Caron et al., 2009; Bik et al., 2012). Community alpha diversity metrics were calculated in mothur and analyzed via ANOVA with *post hoc* Tukey’s test (*p* ≤ 0.05). The Bray–Curtis calculated distance matrices for planktonic and sediment communities were analyzed via permutational MANOVA (perMANOVA) to test for significance in overall differences in community composition between treatments. Bray–Curtis distance matrices were also used for beta diversity analysis and statistical testing using the QIIME (v.1.9.1) make_distance_boxplot.py script (Caporaso et al., 2010). EdgeR was used to detect significant changes in abundances of phylotypes using the edgeR package v 3.12.1 in R (Robinson et al., 2010). Log_2_ ratio of fold-changes (log_2_FC) in abundance of each phylotype were calculated for each fertilized treatment relative to the unenriched treatment. The diversity metric and relative abundance calculations were done on a replicate-by-replicate basis but for the sake of clarity, the visualizations (e.g., **Figures 1, 3, 6, 8**) used replicates pooled by treatment.

Phylotypes are genus-specific taxonomic units identified based on a supervised classification method using reference sequences. However, each phylotype may contain sequences from microbes with differing ecology that can be further resolved based on sequence similarity. Phylotypes of interest were explored further by binning sequences within a phylotype into OTUs. OTU identification is an unsupervised clustering method in which sequences with ≥97% sequence similarity are clustered into the same OTU. Aligned sequences were clustered using the average neighbor method in mothur (Schloss et al., 2009). To construct the maximum-likelihood phylogenetic trees for OTUs from phylotypes of interest (**Figure 5**), reference sequences were identified using the SILVA SINA aligner (Pruesse et al., 2012) and constructed using MEGA 7 (Kumar et al., 2016).

## Results

Here, we summarize the biogeochemical responses to enrichment reported in Lee et al. (2015). After the 21 days of periodic nutrient enrichment, soluble reactive P and total dissolved P concentrations in the water column of fertilized treatments were similar to (P-only and NP16) or significantly lower than (NP75) those in the unenriched control. Seston carbon and chlorophyll *a* concentrations in the water column of NP16 and NP75 treatments increased significantly. In contrast, the P-only treatment experienced marginal changes in measures of biomass. Planktonic (e.g., sestonic) C:P and N:P ratios decreased drastically in all fertilized treatments but were lowest in the P-only treatment; no differences in seston C:N ratios were observed. In the sediment, all treatments had significantly increased total P content (percent of dry mass) but sediment total N content did not change. Microbial cells extracted from sediment had three-fold to four-fold lower C:P and N:P ratios in the NP16 and P-only treatments than in the unenriched control while cells from the NP75 treatment had C:P and N:P ratios similar to the control and cells from all treatments had similar C:N ratios (Lee et al., 2015).

### Bacterial Responses

To assess changes in the relative abundances of bacteria with low versus high rRNA gene copy number as a function of nutrient enrichment in the water column (plankton or planktonic hereafter) communities, planktonic bacterial abundance was assessed by microscopy-based cell counts and qPCR of 16S rRNA gene. The unenriched treatment had both the lowest cell abundances and 16S rRNA gene copies (**Table 1**). With P enrichment, 16S rRNA gene copies mL^-1^ increased significantly and considerably more strongly than did cell counts (14-fold versus 2-fold); thus, 16S rRNA gene copies cell^-1^ (*F*_3,18_ = 18.3, *p*-value < 0.0001) were significantly higher than the control. Both NP16 and NP75 treatments had higher cell abundances and 16S rRNA gene copies ml^-1^ but the 16S rRNA gene copies cell^-1^ were not significantly different from the unenriched treatment.

**Table 1:** Water column 16S rRNA gene copies and cell counts normalized to the sample volume.

Treatment	Cell counts (10^6^ cells mL^-1^± SD)	16S rRNA genes (10^6^ copies mL^-1^ ± SD)	16S rRNA genes (copies cell^-1^ ± SE)
U	0.74 ± 0.13^a^	0.92 ± 0.35^a^	1.29 ± 0.26^a^
P	1.65 ± 0.78^b^	13.35 ± 5.76^b^	9.64 ± 2.67^b^
NP16	5.50 ± 1.50^c^	7.02 ± 2.70^bc^	1.35 ± 0.26^a^
NP75	7.02 ± 1.91^c^	4.29 ± 3.21^c^	0.63 ± 0.19^a^

*The values represent the average of five samples from each treatment. Different letters indicate a significant treatment effect based on ANOVA followed by Tukey’s HSD *post hoc* test.*

**FIGURE 2:**
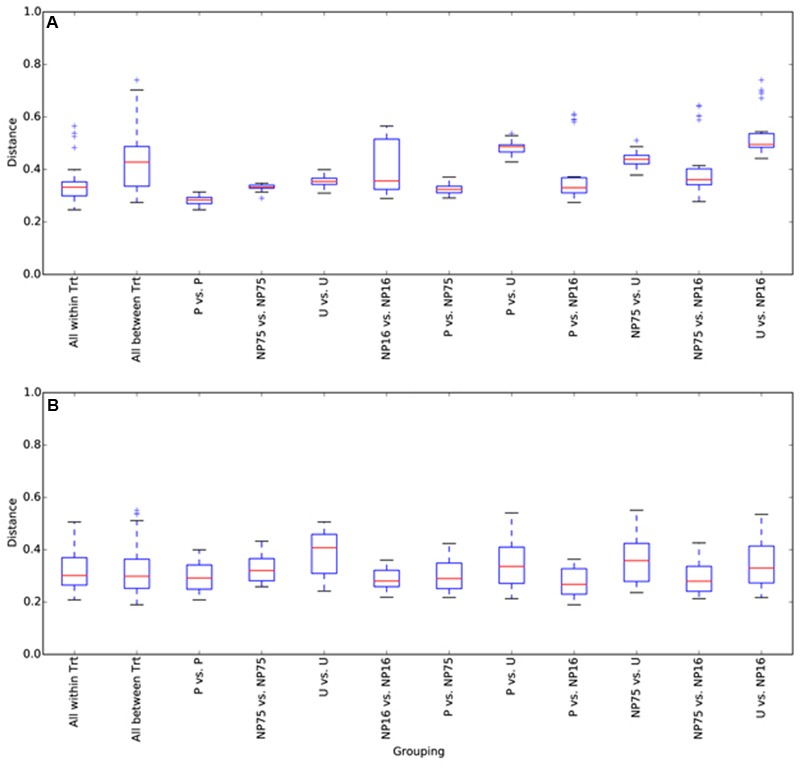
**Box and whisker plots for beta diversity distances of 16S rRNA gene libraries within and between treatments for (A)** water and **(B)** sediment samples. The plot was constructed using the lower and upper quartile of the data (“whiskers” extending from either end of the box; one going from the first quartile to the smallest non-outlier and the other going from third quartile to the largest non-outlier), the inter-quartile range (width of the “box”; the bottom and the top being the lower and upper quartile, respectively), median (red line) and outliers (+ sign). Trt, treatment.

Sequencing of 16S rRNA genes showed significant change in overall planktonic community structure in response to nutrient amendment, based on perMANOVA of the Bray–Curtis distance matrix (*F*_3,12_ = 13.8, *p*-value < 0.001). This change was also evident in the principal coordinates analysis (PCoA) visualization of the Bray–Curtis distance matrix beta diversity (**Figure 1**). Quantitatively, beta diversity was lower within treatments than between treatments. **Figure 2A** shows the distribution of beta diversity when comparing samples within treatment and between treatments. Each treatment exhibited reproducible changes in community composition observed as a tight range of beta diversity (**Figure 2A**). Distribution of beta diversity between treatments was more spread out and had higher values (**Figure 2A**).

**FIGURE 3:**
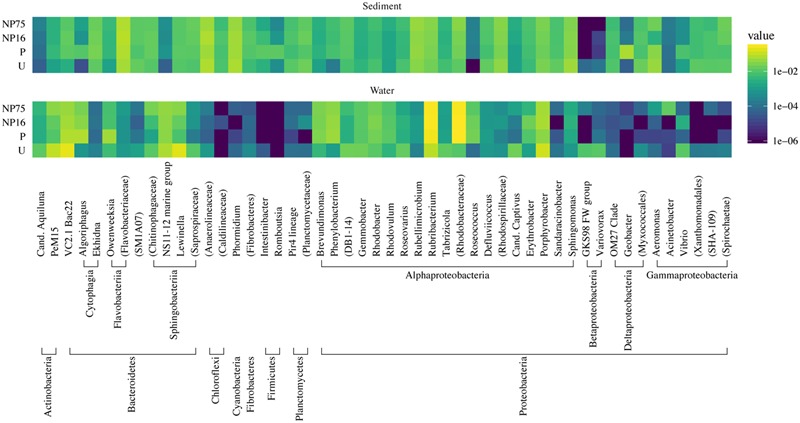
**Taxonomic heat map of the 50 most abundant phylotypes demonstrating the effect of nutrient application on the composition of bacterial communities in the water column and sediment of the mesocosms.** The color scale represents relative abundance as the average percent read totals of five replicates within a treatment, shown in log scale.

In the water column, fertilization (all treatments) significantly decreased community richness (*F*_3,15_ = 15.5, *p*-value < 0.001) but had only minimal effects on evenness and Simpson diversity index (**Table 2**). While there was no significant change in community evenness, the dominant phylotype shifted in all the fertilized treatments. In particular, some of the rare phylotypes (<1% relative abundance) detected in the unenriched treatment decreased to below detection limit in the fertilized treatment while several rare phylotypes became more abundant (**Figure 3**). Across all planktonic samples, the bacterial community was mainly dominated by Proteobacteria and Bacteroidetes, which together made up more than 80% of the population (**Figure 3**). The Proteobacteria consisted primarily of *Porphyrobacter*, a common freshwater bacterium and *Rubribacterium*, an aerobic anoxygenic phototroph. The Bacteroidetes consisted mostly of *Lewinella* and sequences that can only be mapped to the Bacteroidetes VC2.1 Bac22 (**Figure 3**).

**Table 2:** Phylotype-based alpha diversity indices for 16S rRNA gene libraries after normalization to 1038 and 575 sequences for sediment and water libraries, respectively.

Location	Treatment	Shared phylotypes^a^	Observed phylotypes	Chao richness estimator	Simpson evenness	Simpson diversity (1/D)
Water	U	21	66 ± 1.9^a^	118 ± 2.4^a^	0.14 ± 0.03	8.9 ± 1.9
	P	22	50 ± 2.5^b^	90 ± 4.6^b^	0.13 ± 0.01	6.3 ± 0.6
	NP16	18	50 ± 3.8^b^	87 ± 9.8^b^	0.13 ± 0.05	6.5 ± 2.7
	NP75	19	53 ± 4.2^b^	99 ± 12^b^	0.12 ± 0.02	6.4 ± 1.6

Sediment	U	55	199 ± 33	319 ± 47	0.20 ± 0.05	40.4 ± 14.3
	P	67	194 ± 22	317 ± 34	0.16 ± 0.06	31.3 ± 14.0
	NP16	69	211 ± 27	349 ± 45	0.21 ± 0.04	43.6 ± 9.2
	NP75	62	210 ± 38	347 ± 65	0.23 ± 0.02	47.8 ± 12.1

*The values (except for shared phylotypes) represent the average of five samples from each treatment ± SEM. Different letters represent a significant treatment effect based on *post hoc* Tukey’s test. ^a^Number of phylotypes shared between replicates.*

**FIGURE 4:**
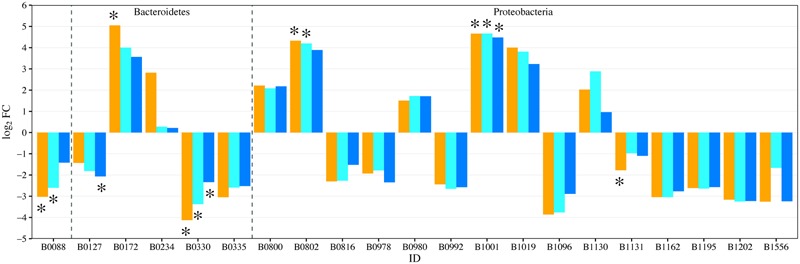
**Fold change (log_2_FC) in bacterial phylotype abundance between U and each of the fertilized treatments (orange = P-only, light blue = NP16, dark blue = NP75).** Only genera with fold change with *p*-value ≤ 0.05 are represented in the figure. ^∗^Represents change with false discovery rate (FDR) ≤ 0.05. B0088: Actinobacteria; Actinobacteria; PeM15 B0127: Bacteroidetes; VC2.1 Bac22 B0172: Bacteroidetes; Cytophagia; Algoriphagus B0234: Bacteroidetes; Flavobacteriia; Owenweeksia B0330: Bacteroidetes; Sphingobacteriia; Lewinella B0335: Bacteroidetes; Sphingobacteriia; uncultured Saprospiraceae B0800: Proteobacteria; Alphaproteobacteria; Brevundimonas B0802: Proteobacteria; Alphaproteobacteria; Phenylobacterium B0816: Proteobacteria; Alphaproteobacteria; DB1-14 B0978: Proteobacteria; Alphaproteobacteria; Roseovarius B0980: Proteobacteria; Alphaproteobacteria; Rubribacterium B0992: Proteobacteria; Alphaproteobacteria; Tabrizicola B1001: Proteobacteria; Alphaproteobacteria; uncultured Rhodobacteraceae B1019: Proteobacteria; Alphaproteobacteria; Roseococcus B1096: Proteobacteria; Alphaproteobacteria; Candidatus Captivus B1130: Proteobacteria; Alphaproteobacteria; Erythrobacter B1131: Proteobacteria; Alphaproteobacteria; Porphyrobacter B1162: Proteobacteria; Betaproteobacteria; GKS98 freshwater bacteria B1195: Proteobacteria; Betaproteobacteria; Polaromonas B1202: Proteobacteria; Betaproteobacteria; Variovorax B1556: Proteobacteria; Gammaproteobacteria; Vibrio

**FIGURE 5:**
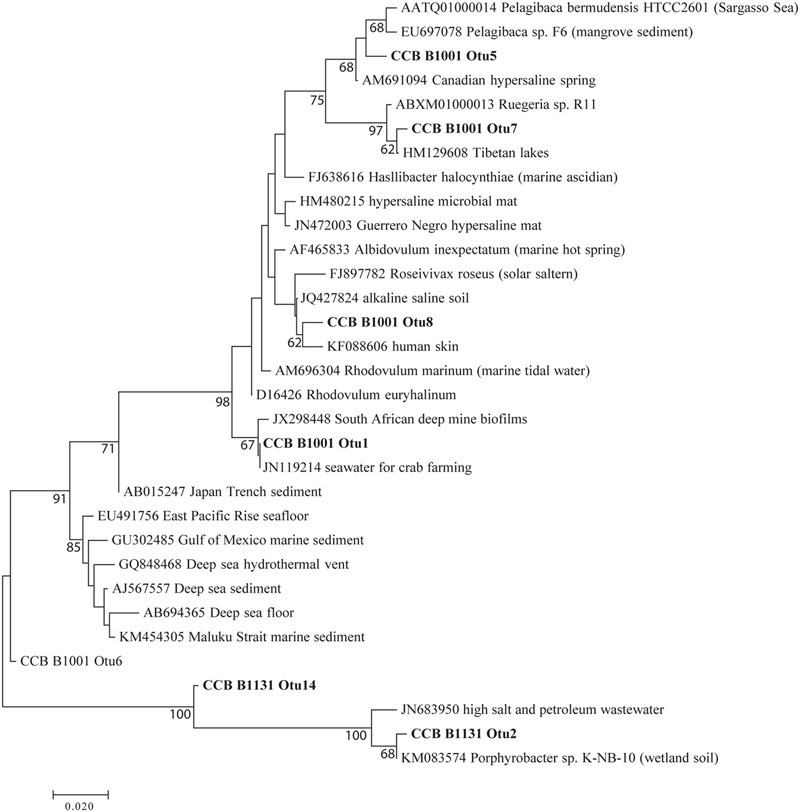
**Maximum-likelihood phylogenetic tree of planktonic Alphaproteobacteria B1001**.

EdgeR analysis identified two phylotypes that were significantly affected in the water column of all three fertilized treatments (**Figure 4**). Significance was defined as log_2_FC with a false discovery rate (FDR) or 5% or less. *Lewinella* (B0330) significantly decreased in all three fertilized treatments by more than four-fold while an uncultured member of the Rhodobacteraceae (B1001) increased in dominance in all three fertilized treatments (**Figure 4**). The enriched Rhodobacter had a relative abundance of 1% or less in the unenriched treatment but contributed over 20% in all the fertilized treatments (**Figure 3**). The OTUs within this phylotype are most phylogenetically similar to Rhodobacteraceae of marine origin (**Figure 5**).

Relative to controls, the P-only and NP16 treatments also displayed a significant decrease in Actinobacteria PeM15 sequences (B0088) while *Phenylobacterium* (B0802) sequences increased. The P-only treatment also experienced a significant enrichment of *Algoriphagus* (B0172) with a log_2_FC of 5.05. Both *Phenylobacterium* and *Algoriphagus* were present at less than 1% in the unenriched treatment. We also note that *Porphyrobacter* (B1131) and Bacteroidetes VC2.1 (B0127), two dominant phylotypes in the unamended treatment, were negatively affected by nutrients, especially in the P-only and NP75 treatments, respectively.

In the surficial sediment, no significant changes in the community structure were detected based on analysis of the Bray–Curtis distance between the treatments (perMANOVA *p*-value > 0.05). PCoA visualizations of beta diversity similarly failed to show reproducible community patterns (**Supplementary Figure S1**). Quantitatively, the replicates within each treatment in the sediment had similar beta diversity in comparison with samples from different treatments, even without nutrient amendment. Essentially, each spatial replicate was already quite different from each of the others. This observation is supported by comparison of the distribution of beta diversity between and within treatment for water (**Figure 2A**) and sediment (**Figure 2B**) samples. The ranges of distances for sediment samples between and within treatment are similar, while the water samples showed a larger range of beta diversity between treatments than within treatment. Alpha diversity measures of the sediment bacterial community also did not appear to be significantly affected by nutrient enrichment (**Table 2**). As expected, the sediment bacterial community was more diverse than the planktonic community, with over three times as many phylotypes (**Table 2**). Phototrophs such as Chloroflexi and Cyanobacteria were more dominant in the sediment with relative abundance of 4.5–6.3% and 4.2–5.8%, respectively (**Figure 3**).

### Eukaryotic Responses

**FIGURE 6:**
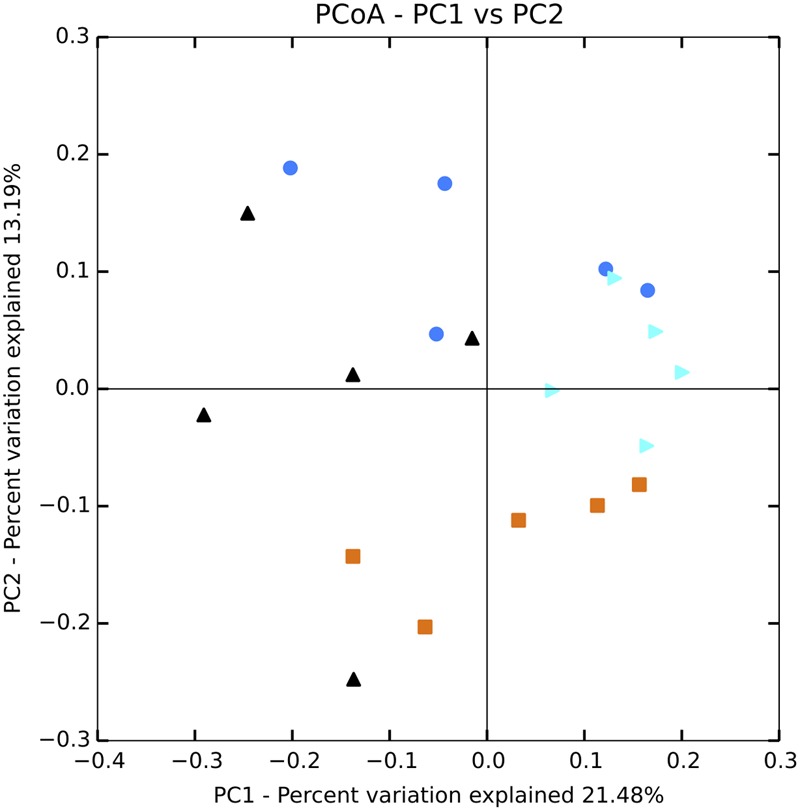
**Principal coordinates analysis plot of Bray–Curtis distances for eukaryote communities in the water column.** Black = unenriched, orange = P-only, light blue = NP16, dark blue = NP75.

**FIGURE 7:**
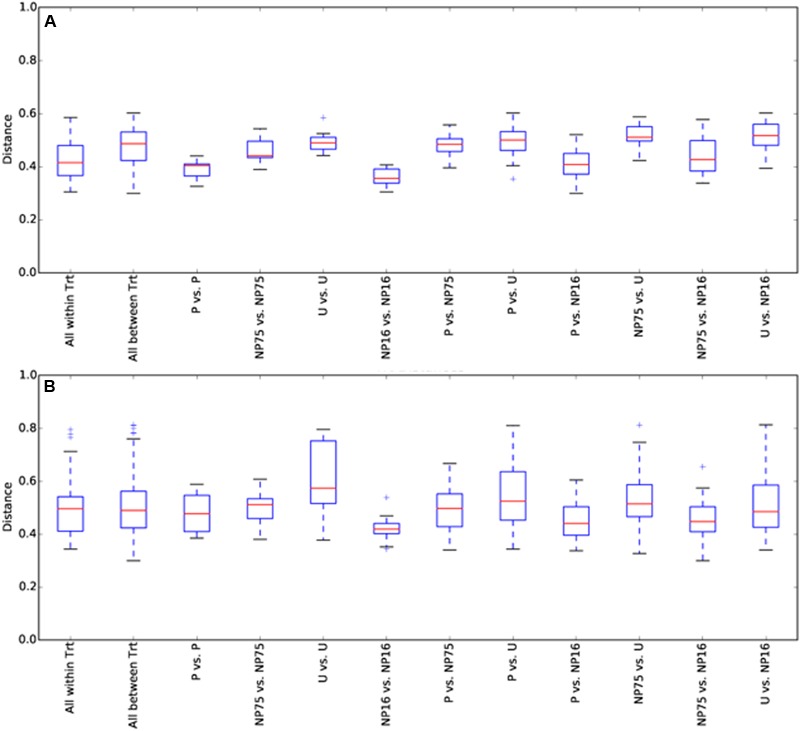
**Box and whisker plot for beta diversity distances of 18S rRNA gene libraries within and between treatments for (A)** water and **(B)** sediment samples. The plot was constructed using the lower and upper quartile of the data (“whiskers” extending from either end of the box; one going from the first quartile to the smallest non-outlier and the other going from third quartile to the largest non-outlier), the inter-quartile range (width of the “box”; the bottom and the top being the lower and upper quartile, respectively), median (red line) and outliers (+ sign). Trt, treatment.

Planktonic eukaryotic community structure among replicates within a treatment was also highly variable but a significant change in community structure (perMANOVA: *F*_3,12_ = 9.59, *p*-value = 0.001) was still apparent (**Figure 6**). Nutrient enrichment tended to decrease phylotype richness, although this effect was not significant due to large variation within treatments (**Table 3**). The high variability between replicates was captured in the beta diversity analysis. Nevertheless, beta diversity of the planktonic communities between treatments was still higher than within treatment (**Figure 7A**).

**Table 3:** Alpha diversity indices for 18S rRNA gene libraries.

Location	Treatment	Shared phylotypes^a^	Observed phylotypes	Chao richness estimator	Simpson evenness	Simpson diversity (1/D)
Water	U	16	60 ± 14	71 ± 15	0.05 ± 0.01	3.20 ± 0.88
	P	18	50 ± 12	64 ± 16	0.05 ± 0.01	2.57 ± 0.37
	NP16	16	36 ± 5.2	48 ± 4.8	0.08 ± 0.01	2.70 ± 0.28
	NP75	13	43 ± 14	59 ± 21	0.07 ± 0.04	2.56 ± 0.37

Sediment	U	12	89 ± 19	103 ± 20	0.10 ± 0.08	9.56 ± 8.71
	P	15	86 ± 24	105 ± 28	0.08 ± 0.05	6.07 ± 3.27
	NP16	30	83 ± 10	101 ± 11	0.06 ± 0.03	4.95 ± 1.74
	NP75	16	69 ± 16	82 ± 19	0.07 ± 0.03	4.28 ± 1.35

*The libraries were normalized to 10,521 and 9,299 sequences for sediment and water libraries, respectively. The values represent the average of five samples from each treatment ± SEM. ^a^Number of phylotypes shared between replicates.*

**FIGURE 8:**
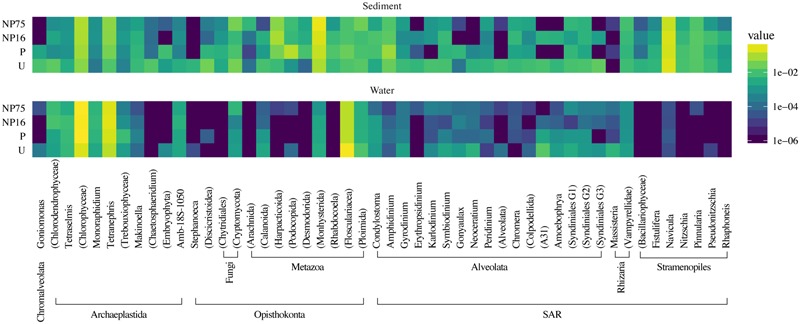
**Taxonomic heat map of the 50 most abundant phylotypes demonstrating the effect of nutrient application on the composition of eukaryotic communities in the water column and sediment of the mesocosms.** The color scale represents relative abundance as the average percent read totals of five replicates within a treatment, shown in log scale.

**FIGURE 9:**
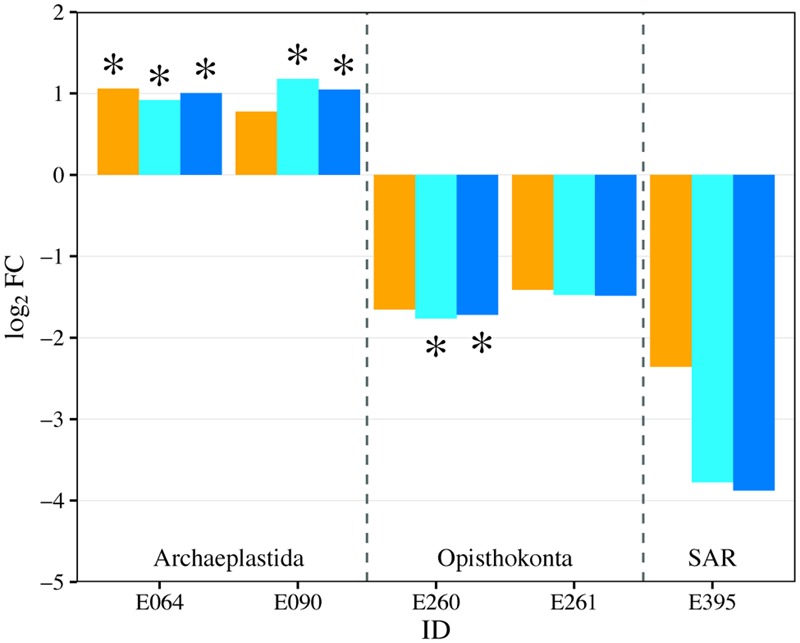
**Fold change (log_2_FC) in eukaryotic genera abundance between the control treatment (U) and each of the fertilized treatments (orange = P-only, light blue = NP16, dark blue = NP75).** Only genus with fold change that has *p*-value ≤ 0.05 is represented in the figure. ^∗^Represents change with FDR ≤ 0.05. E064: Archaeplastida; Chloroplastida; Tetranephris E090: Archaeplastida; Chloroplastida; unclassified E260: Opisthokonta; Metazoa; Flosculariacea E261: Opisthokonta; Metazoa; Ploimida E395: SAR; Alveolata; A31

Planktonic eukaryotic community was highly uneven with two phylotypes of green algae (Chlorophyceae) and a rotifer (Flosculariacea) making up over 80% of the 18S rRNA gene sequence library (**Figure 8**). Nonetheless, EdgeR analysis identified three phylotypes that were significantly responsive to nutrient addition (either P alone or N and P at both ratios) in the water column (**Figure 9**). Two green alga OTUs (E064, E090) significantly increased in dominance in the fertilized treatments while a rotifer (E260) was negatively affected by nutrient addition. Indeed, the two-fold increase in the green algae led to this group contributing more than 90% of the 18S sequence library in the fertilized treatments. It should also be noted that the protist Perkinsidae (E395) was present between 0.4 and 6.3% in the unenriched treatment but decreased to below detection in most of the mesocosms receiving both N and P (**Figures 8, 9**).

In the sediment, no distinct eukaryotic community structure was observed for different treatments, with all the samples exhibiting high beta diversity (**Figure 7B**) and similar measures of alpha diversity (**Table 3**). Hence, no clustering of samples from the same treatment is observed in the PCoA plot (**Supplementary Figure S2**). Sediment eukaryotic community consisted mostly of benthic diatoms, nematodes, and dinoflagellates (**Figure 8**). EdgeR analysis also did not identify any eukaryotic sediment phylotypes that were significantly influenced by nutrient enrichment.

## Discussion

The CCB hosts diverse water bodies with unique microbiota (Souza et al., 2006) and it has been hypothesized that low phosphorus availability itself maintains high diversity of microorganisms, some potentially endemic, in the basin (Souza et al., 2008). In this study, we evaluated the effects of nutrient stoichiometry on both the bacterial and eukaryotic community in P-deficient Lagunita Pond by conducting an *in situ* mesocosm experiment. Most freshwater studies investigating the effects of nutrients focus on nutrient availability *per se* but the ratio of available nutrients can also play an important role in shaping the microbial community (Elser et al., 2003; Hessen et al., 2013). Based on the GRH, we expected that phylotypes that are responsive to P enrichment would have high rRNA gene copy number that allows high ribosome production capacity. We also sought to determine if the N:P ratio of nutrient enrichment itself was important in shaping microbial responses and driving community structure.

Nutrient enrichment, either P alone or both N and P at two different ratios, altered community structure as shown in the beta diversity analysis but had minimal effect on alpha diversity of the microbial community. In particular, there was a significant change in the structure of Lagunita’s planktonic microbial community, enriching for phylotypes that were rare in the unenriched treatment. P addition (without N) resulted in the highest number of significantly affected bacterial phylotypes followed by enrichment at 16:1 and 75:1. This indicates that the ratio of the added nutrient plays an important role in shaping the planktonic microbial community in this system. The extent of change in community structure is also congruent with previously observed changes in the N:P ratio of these treatments, where P-only mesocosms experienced the largest change in dissolved N:P ratio (Lee et al., 2015). We suspect that only the P-alone treatment sufficiently reduced the overall N:P ratio to levels where P was no longer limiting; enrichment at 16:1 and (especially) 75:1 ratios likely did not eliminate P-limitation given the extremely high ambient N:P ratios characteristic of Lagunita Pond.

Importantly, we found a significant nutrient enrichment effect on 16S rRNA gene copy number per cell (**Table 3**) in the P-only treatment. While the absolute magnitudes of the rRNA gene copy number per cell values we report likely have some associated error due to uncertainties in cell counts and PCR (e.g., primer coverage), these uncertainties likely held across all treatments and thus should not impact among-treatment comparisons. The closest available reference genomes for responsive phylotypes in the P-only treatment were analyzed for ribosome production capability, and each have rRNA gene copy number between 1 and 3 copies. *Algoriphagus* sp., which is highly enriched in the P-only treatment had the highest rRNA gene copy number of 3 (Stoddard et al., 2014). However, the majority of phylotypes in the samples are not very well characterized. Thus, it is likely that the reference genomes are simply not appropriate for inferring the rRNA copy number of related microbes in nature, especially in a poorly studied and unique environment such as Cuatro Cienegas. Overall, the data provide some of the first field evidence supporting the hypothesized connection between P supply and ribosomal RNA genome organization under the GRH and suggests that P enrichment at low N:P ratio favors bacteria with higher rRNA gene copy number. The result is congruent with previous work investigating ecological strategies of bacteria with low and high rRNA gene copy numbers (Klappenbach et al., 2000; Nemergut et al., 2016). It also warrants further investigation of the rRNA gene copy numbers of the other positively responding phylotypes that have yet to be classified. The lack of significant change in the rRNA gene copy number per cell in the NP16 and NP75 treatments compared to the unenriched treatment may indicate that growth of bacteria with high rRNA gene copy number remained limited by P as argued above. Moreover, NP75 treatment may even have increased P limitation in relation to N because both sediment and seston N:P ratios in the NP75 treatments were still at least twice the Redfield ratio (Lee et al., 2015).

Given that there were only two phylotypes that responded uniquely in the P-only treatment, change in community structure may not be a sufficient explanation for the increased rRNA gene copies/cell observed in the P-only treatment. An alternative explanation is that the high rRNA gene copy number members have a higher growth rate in the P-only treatment and thus contain more copies per cell due to ongoing DNA replication in each cell. The method used to measure rRNA gene content does not distinguish cells with higher rRNA gene copy number in the genome from cells with higher rRNA gene content due to multiple DNA replication forks. At a high growth rate, cells initiate DNA replication prior to cell division, creating multiple replication forks that consequently increase the effective copy number of genes located near the origin of replication (*oriC*) by up to eight-fold (Skarstad et al., 1986). Highly transcribed genes involved in transcription and translation, such as rRNA genes, are often found to be in close proximity to the *oriC*, especially in fast-growing bacteria (Couturier and Rocha, 2006; Vieira-Silva and Rocha, 2010). If the responsive phylotypes have two copies of rRNA operons near the origin of replication, the measured rRNA gene content/cell could increase by another two-fold. We cannot resolve this question with the data we currently have available.

Variation in organismal stoichiometry of microbial eukaryotes, especially phytoplankton, has also been attributed to the allocation of resources to growth machinery (i.e., ribosomes) vs. resource acquisition machinery (i.e., chloroplasts) (Arrigo, 2005; Klausmeier et al., 2007). The planktonic eukaryotic community response pattern was more representative of a general nutrient limitation. The stimulation of green algae we observed is consistent with the observed increase in chl *a* concentration in the fertilized treatments (Lee et al., 2015). A previous study in the CCB region found that P enrichment enhanced the proliferation of diatoms rather than green algae (Elser et al., 2005a). Diatoms are also expected to be more successful in environments with low N:P regime (Arrigo, 2005). However, most of the diatoms in Lagunita are found in the sediment, where they might have had limited accessibility to the added phosphorus.

No statistically significant changes in the microbial community were observed in the sediment in response to nutrient enrichment. However, it cannot be concluded that sediment microbial communities are resistant to nutrient perturbation because highly variable community structure was observed for all the samples (**Figures 2, 7**). The high variation in the sediment community is not surprising due to high heterogeneity in the local habitat (Fenchel, 2002). The evaporitic nature of Lagunita further increases habitat heterogeneity, which can lead to high variation in community structure as the communities recover independently (Shade et al., 2008). The high diversity between the different samples within each treatment highlights the critical need for replication in microbial community analysis, especially for field samples.

Further investigation targeting the responsive phylotypes will be required to determine other mechanisms of response elicited by changes in nutrient ratio. While the qPCR results from the P-only treatment supports the GRH, it is unlikely to be the only biochemical/physiological response to alterations in nutrient supply ratios. In fact, the GRH was not supported in another study exploring the large range of rRNA gene copy number in *Bacillus* sp. isolated from CCB (Valdivia-Anistro et al., 2015). Consistent with their study, *Bacillus* sp., which was found to be less than 0.5% of the sequence libraries, did not exhibit any treatment effect (data not shown). Although *Bacillus* sp. is not a dominant phylotype in CCB, the use of cultivation-dependent methods in Valdivia-Anistro et al. (2015) suggests that species with high rRNA gene copy number can survive and remain active in such a P-limited environment. In fact, the discovery of cultivable *Bacillus* sp. in CCB at different times suggests that high rRNA gene copy number may be a more important trait for rapid response to changes in rapidly fluctuating physical factors, such as moisture. Furthermore, Ponce-Soto et al. (2015) found that antibiotic resistance decreased in the Lagunita bacterial community after nutrient enrichment. Decreases in antibiotic resistance suggest that antagonistic interactions between microbes decrease, which may create new niche space for the rare phylotypes. Antagonistic activity was found to be more common for particle-associated bacteria (Long and Azam, 2001). The enrichment with dissolved nutrient may have selected for free-living species that are more efficient at taking up the added nutrient and have less antagonistic activity. One particular phylotype that warrants further investigation is the appearance of uncultured Rhodobacteraceae in the fertilized treatment, while *Rubribacterium* was the sole dominant Rhodobacteraceae in the unenriched treatment.

It was previously discovered that a large number of bacterial phylotypes in CCB are closely related to bacteria of marine origin (Souza et al., 2006). We note that the planktonic bacterial community in Lagunita is highly enriched with aerobic anoxygenic phototrophic (AAP) bacteria. High AAP abundance is typical of oligotrophic temperate lakes, especially during the summer (Koblížek, 2015). The importance of light-sensing mechanisms in CCB was previously suspected when *Bacillus coahuilensis* was found to carry a constitutive bacteriorhodopsin gene (Alcaraz et al., 2011). Phototrophic metabolism provides a competitive advantage in the macrophyte-free Lagunita, where dissolved organic carbon sources are limited while the use of light as energy source allows AAP bacteria to have higher growth efficiency compared to heterotrophs (Koblížek, 2015). Unlike most freshwater ecosystems where the phylum Actinobacteria is often the most abundant (Newton et al., 2011; Ghai et al., 2012), Alphaproteobacteria and Bacteroidetes dominated the Lagunita water column. Bacteroidetes are known for their ability to degrade secondary metabolites from eukaryotes and hence have a dynamic relationship with eukaryotic primary producers (Eiler and Bertilsson, 2004; Schauer et al., 2005). Carbon cycling in Lagunita is likely driven by eukaryotic phototrophs but their relationship with the Bacteroidetes remains to be elucidated because nutrient addition had opposing effects on these two groups of microorganisms. Lagunita is also dominated by non-Flavobacteriales Bacteroidetes that are not commonly found in freshwater ecosystems. For example, phylotype B0127 can only be mapped to Bacteroidetes VC2.1 Bac22, which consists of sequences obtained mostly from marine environments such as hydrothermal vents, marine snow, and alkaline lakes (Zhang et al., 2013). The physiology of this cluster remains to be investigated because there is still no genomic or cultured representative. In addition, the dominant Actinobacteria in Lagunita is a more closely related to the marine PeM15 than to the more common acl1 lineage (Hahn, 2009). The nutrient-responsive uncultured Rhodobacteraceae phylotype also consists of sequences that are closely related to sequences obtained from marine ecosystems. Further investigation targeting these unique phylotypes will be required to understand how some of these phylotypes persist under P-limitation and strong stoichiometric imbalance.

## Perspectives and Conclusion

Our study is among the first to use molecular surveys within a replicated field experiment to evaluate how the bacterial and microbial eukaryotic communities of a severely- P-limited ecosystem respond to perturbation of supplies and ratios of key nutrients. Our data highlight the importance of marine-affiliated taxa such as Bacteroidetes cluster VC2.1 Bac22 and uncultured Rhodobacteraceae in support of the surprising inference of Souza et al. (2006) that CCB’s biological history has retained a signature of its ancient geologic marine history. High-throughput sequencing of both the bacterial and eukaryotic community further confirmed that CCB is rich in microbial biodiversity and especially in major phylotypes that are not well-described. At the community level, our results suggest that nutrient enrichment had relatively modest impacts on overall taxonomic diversity but led to significant shifts in community structure, particularly in the water column. This provides further evidence that nutrient ratios play an important role in shaping the structure of both bacterial and eukaryotic communities. Finally, we provide evidence that, contributing to these nutrient-driven community shifts, are changes in the abundances of taxa that differ in rRNA gene copy number, consistent with the GRH. Future studies using metagenomic approaches coupled to *in situ* nutrient enrichment may shed further light on the underlying mechanisms, and functional consequences, of nutrient enrichment in oligotrophic and stoichiometrically imbalanced ecosystems.

## Author Contributions

ZL was involved in conducting the sampling, analyzing samples, analyzing the data, and writing the manuscript. JS was involved in designing the study conducting the sampling, analyzing the data, and writing the manuscript. AP-P was involved in analyzing samples, analyzing the data, and writing the manuscript. DK was involved in analyzing the data. AM was involved in analyzing the data. AA was involved in analyzing the data. CD was involved in designing the data analysis plan, analyzing the data, and writing the manuscript. LE was involved in designing the study, conducting the sampling, and writing the manuscript. VS was involved in designing the study, conducting the sampling, analyzing the data, and writing the manuscript. JE was involved in envisioning the original study, designing the experiment, conducting the sampling, advising on data analysis, and writing the manuscript.

## Conflict of Interest Statement

The authors declare that the research was conducted in the absence of any commercial or financial relationships that could be construed as a potential conflict of interest.

## Supplementary Material

FIGURE S1 **PCoA plot of Bray–Curtis distances for bacterial communities in the sediment.** Black = unenriched, orange = P-only, light blue = NP16, dark blue = NP75.

FIGURE S2 **PCoA plot of Bray–Curtis distances for eukaryotic communities in the sediment.** Black = unenriched, orange = P-only, light blue = NP16, dark blue = NP75.
